# Why is timing of bird migration advancing when individuals are not?

**DOI:** 10.1098/rspb.2013.2161

**Published:** 2014-01-07

**Authors:** Jennifer A. Gill, José A. Alves, William J. Sutherland, Graham F. Appleton, Peter M. Potts, Tómas G. Gunnarsson

**Affiliations:** 1 School of Biological Sciences, University of East Anglia, Norwich Research Park, Norwich NR4 7TJ, UK; 2 Conservation Science Group, Department of Zoology, University of Cambridge, Downing Street, Cambridge CB2 3EJ, UK; 3 British Trust for Ornithology, The Nunnery, Thetford, Norfolk IP24 2PU, UK; 4 Farlington Ringing Group, Solent Court Cottage, Chilling Lane, Warsash, Southampton SO31 9HF, UK; 5 South Iceland Research Centre, University of Iceland, Bankavegi, IS-800 Selfoss and Gunnarsholt, IS-851, Hella, Iceland

**Keywords:** migratory behaviour, phenology, population change, shorebird, timing of breeding

## Abstract

Recent advances in spring arrival dates have been reported in many migratory species but the mechanism driving these advances is unknown. As population declines are most widely reported in species that are not advancing migration, there is an urgent need to identify the mechanisms facilitating and constraining these advances. Individual plasticity in timing of migration in response to changing climatic conditions is commonly proposed to drive these advances but plasticity in individual migratory timings is rarely observed. For a shorebird population that has significantly advanced migration in recent decades, we show that individual arrival dates are highly consistent between years, but that the arrival dates of new recruits to the population are significantly earlier now than in previous years. Several mechanisms could drive advances in recruit arrival, none of which require individual plasticity or rapid evolution of migration timings. In particular, advances in nest-laying dates could result in advanced recruit arrival, if benefits of early hatching facilitate early subsequent spring migration. This mechanism could also explain why arrival dates of short-distance migrants, which generally return to breeding sites earlier and have greater scope for advance laying, are advancing more rapidly than long-distance migrants.

## 1. Introduction

Changes in the timing of spring migration have been widely reported for many species in recent decades [1–6], and advances in migration are among the most commonly reported phenological responses to climatic change [1,7]. As early arrival on the breeding grounds has been linked to improved individual fitness [8–12] and as early arrival risks the costs of harsh weather and low-resource abundance, it is likely that individual migratory timings are under strong selection pressure [13,14].

The recent advances in timing of migration have been linked to changes in climatic conditions but the mechanisms driving shifts in timing of migration are unknown [7]. Identifying these mechanisms is critically important because population declines are being most widely recorded in species that are not advancing migration [15]. The most commonly proposed mechanism explaining the observed shifts in timing is individual plasticity in timing of migration [4,7,16]. This mechanism is often proposed because species that migrate over shorter distances are frequently reported to have advanced more than longer distance migrants [2,5,17]. This pattern suggests that long-distance migrants may be less capable of responding to changing conditions at their destination, because the greater distance reduces their capacity to predict conditions on the breeding grounds and/or because these species have stronger endogenous control of migration timing [7,18]. However, shifts in timing are typically recorded at the population level, with most studies reporting first or mean arrival dates at a given location [5,7]. Identifying the role of individual plasticity in driving population-level advances in timing requires repeated measurement of individual arrival dates over multiple migration periods.

The dates of spring arrival into Iceland of a wide range of migratory bird species have advanced significantly over the last two decades and these advances have coincided with rising temperatures [6]. Among these species, the first arrival dates of Icelandic black-tailed godwits, *Limosa limosa islandica*, have advanced significantly, at rates similar to other short-distance (within-continent) migrants [6]. Icelandic black-tailed godwits are also the focus of a unique long-term, population-wide study in which individuals have been marked and tracked throughout the migratory range by a network of more than 2000 volunteer observers. We use a 14 year dataset of arrival dates of marked individuals of this population to quantify annual variation in individual migratory timings and investigate the mechanisms driving shifts in migratory timings.

## 2. Material and methods

### (a) Individual arrival dates

The Icelandic black-tailed godwit population numbers approximately 50 000 individuals [19] and, over the last two decades, colour-ringing throughout the breeding and winter ranges has been used to maintain approximately 1–2% of the population individually identifiable in the field [19,20]. Since 1999, regular, repeated surveys every 1–3 days of the main spring arrival locations in Iceland have been undertaken throughout the arrival period (details in [21]), during which the arrival dates of individually marked birds are recorded. Arrival sites are estuarine mudflats and coastal wetlands, and all individuals present on these sites can typically be observed (median proportion of birds in arrival flocks checked for colour rings = 0.99, mean = 0.84±0.02 s.e.). Between 1999 and 2012, arrival dates for 54 individuals were recorded in between four and eight springs (see the electronic supplementary material). Most godwits are caught and ringed as adults, and are therefore of unknown age. However, arrival dates have been recorded for 46 individuals that were either ringed as chicks in Iceland or during their first winter, and are therefore of known age. Of these 46 individuals, 38 have been recorded on arrival in 1 year, six in 2 years and two in 3 years.

### (b) Estimating laying dates and intervals between arrival and laying

Since 2001, approximately 100 nests per season of lowland-breeding waders (black-tailed godwit *Limosa limosa*, snipe *Gallinago gallinago*, redshank *Tringa totanus*, golden plover *Pluvialis apricaria*, oystercatcher *Haematopus ostralegus* and whimbrel *Numenius phaeopus*) have been located through intensive surveys of study locations throughout south Iceland. The laying date of each nest is estimated by conventional egg-floating techniques [22] and, in many cases, confirmed by recording hatching date. To compare advances in arrival among species with differing times of arrival and nest-laying, the first known date of nest initiation for each of these species (between 34 and 161 nests per species) was extracted from these data and used to calculate the time interval (days) between arrival and laying for each species. The arrival date for each species was the average first arrival date reported in [6]. The advance in timing of arrival for each species was extracted from [6] and was the slope of regressions of annual variation in first date of arrival between 1988 and 2009.

For black-tailed godwits, repeatability of laying dates was estimated for nests of marked individuals, as many of these nesting events have one or both parents individually marked (having either been caught elsewhere in the range or during a previous nesting attempt). Repeatability of laying dates is estimated here for females; few mate changes have occurred as godwits are strongly mate-faithful [20].

### (c) Estimating annual variation in hatch dates

Since 1999, over 740 black-tailed godwit chicks (average per year = 57 ± 34.2 s.d.) have been caught at locations throughout Iceland, between mid-June and mid-July. Chicks are individually colour-ringed and biometrics are recorded. To assess annual variation in godwit hatch dates, mean relative chick size (the residual variation from the relationship between total head length and day (ordinal date) of ringing) was calculated for each year, as chicks will be larger on any given day in years in which hatching is earlier.

### (d) Weather data

Mean monthly temperature data were extracted for two weather stations of the Icelandic Meteorological Office (www.vedur.is). Mean April temperatures at Reykjavík (64°07′ N, 21°54′ W), a major arrival location, were used to explore the influence of temperature on spring arrival dates. Mean June temperatures at Hæll (64°3.9′ N, 20°14.5′ W), the closest station to the breeding study locations, were used to explore the influence of temperature on nest-laying dates. In addition, values of the North Atlantic Oscillation (NAO) index were extracted for March and April (http://www.cru.uea.ac.uk/cru/data/nao/), the months prior to and during migration.

### (e) Statistical analyses

Annual, individual/pair and temperature-related variation in arrival and laying dates (both measured as ordinal dates, from 1 January) were explored using general linear models (GLMs) with normal error distributions, with year or temperature fitted as covariates and individual or pair as fixed factors. Annual variation in arrival dates of known-age individuals and in mean relative chick size were explored with GLMs with year fitted as a covariate. Repeatability of individual arrival dates between years was calculated following [23].

## 3. Results

### (a) Population and individual spring arrival dates

**Figure 1.**
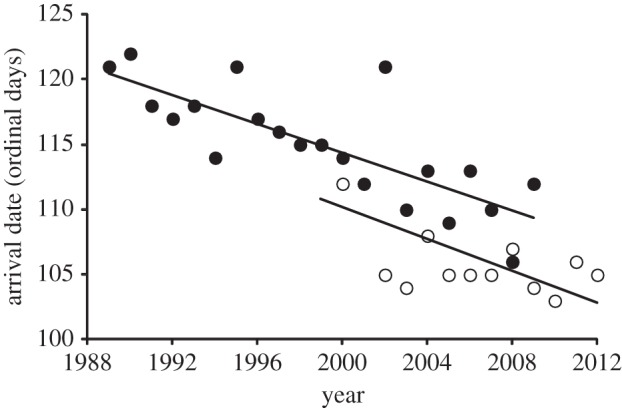
The annual change in first spring arrival date of black-tailed godwits in south Iceland reported by Gunnarsson & Tómasson [6] (closed circles: *y* = −0.55*x* + 1221, *r*^2^ = 0.62, *n* = 21, *p* < 0.001), and the date on which the first individually marked black-tailed godwits were recorded on passage sites in Iceland (open circles: *y* = −0.61*x* + 1332, *r*^2^ = 0.5, *n* = 14, *p* < 0.005).

**Figure 2.**
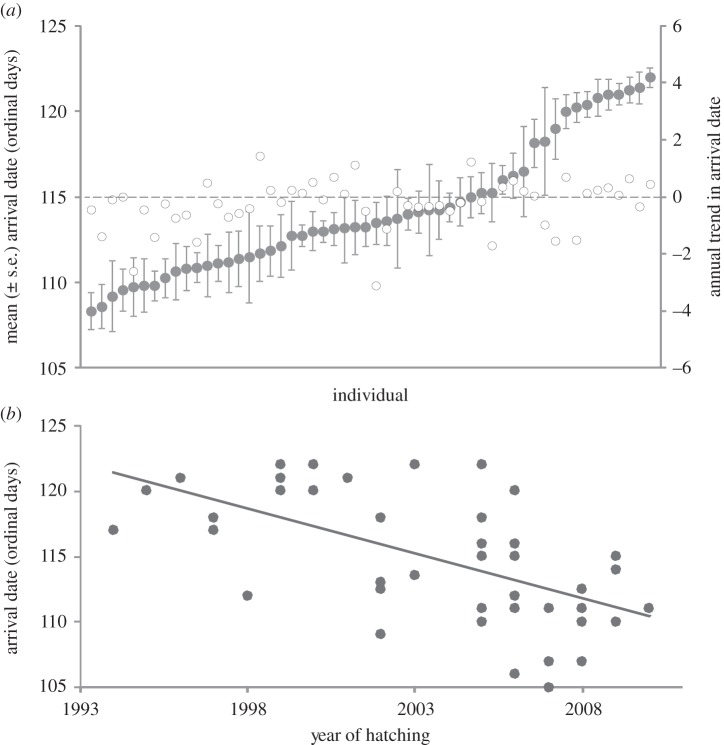
Dates of spring arrival into Iceland of (*a*) 54 individually marked black-tailed godwits recorded on arrival in between 4 and 8 years, from 1999 to 2012 (filled circles, ordered from earliest to latest) and the rates of change (open circles, days per year) in arrival among these individuals and (*b*) 46 individuals hatched in different years (*y* = 1496–0.69*x*, *r*^2^ = 0.34, *p* < 0.001), and subsequently recorded on spring arrival.

The timing of arrival of black-tailed godwits in Iceland has advanced significantly, as shown by the date on which the first marked individuals have been recorded at coastal arrival sites since 1999, and the date of the first godwit arrival at an inland site in Iceland since 1989 (figure 1). However, despite these population-level advances in timing of arrival, repeated measurement of individual arrival dates over multiple years shows that individuals are highly consistent in their dates of arrival (repeatability = 0.51, *F*_53,230_ = 6.6, *p* < 0.001; figure 2*a*). There is no significant trend in individual arrival dates over time (figure 2*a*; trends distributed above and below zero; table 1*a*) and individual arrival dates are not significantly earlier in warmer springs (table 1*a*).

**Table 1.** Results of GLMs of annual and individual variation in (*a*) spring arrival dates of 54 Icelandic black-tailed godwits (4–8 years between 1999 and 2012) and in relation to mean April (the main arrival month) temperatures (°C) in Iceland, and (*b*) laying dates for 15 pairs of godwits recorded in 2–3 years between 2001 and 2012 and in relation to mean June (the main nesting month) temperatures (°C) in south Iceland. (Significant effects are highlighted in italics.)

	d.f.	*F*	*p*	parameter (±s.e.)
(*a*)				
year	1	2.69	0.102	−0.13 (±0.08)
individual	53	5.31	<*0.001*	
error	229			
temperature	1	1.76	0.186	0.23 (±0.17)
individual	53	6.44	<*0.001*	
error	229			
(*b*)				
year	1	4.78	*0.042*	−2.55 (±1.17)
pair	14	1.31	0.289	
error	18			
temperature	1	1.76	*0.018*	−4.04 (±1.55)
pair	14	6.44	0.446	
error	229

If individuals are consistent in their migratory timings but populations are advancing, the advances must result from new recruits migrating earlier, on average, than recruits from earlier years. Arrival dates of individual godwits hatched in recent years are indeed significantly earlier than arrival dates of their predecessors (figure 2*b*). Arrival dates do not appear to change with age, as three of these known-age individuals were first recorded in their second calendar year (the likely recruitment year), and the average difference between their arrival dates in this year and subsequent years was 1 day for two individuals seen in 2 years and 3 days for one individual seen in 3 years.

### (b) Potential drivers of changes in recruit arrival dates

Identifying the drivers of advances in the arrival dates of recruits is key to understanding the links between changes in migration timing and population status. Several mechanisms could potentially alter recruit timings, and thus the frequency of individuals with different migratory timings, within this population.

#### (i) Carry-over effects of changing natal conditions

**Figure 3.**
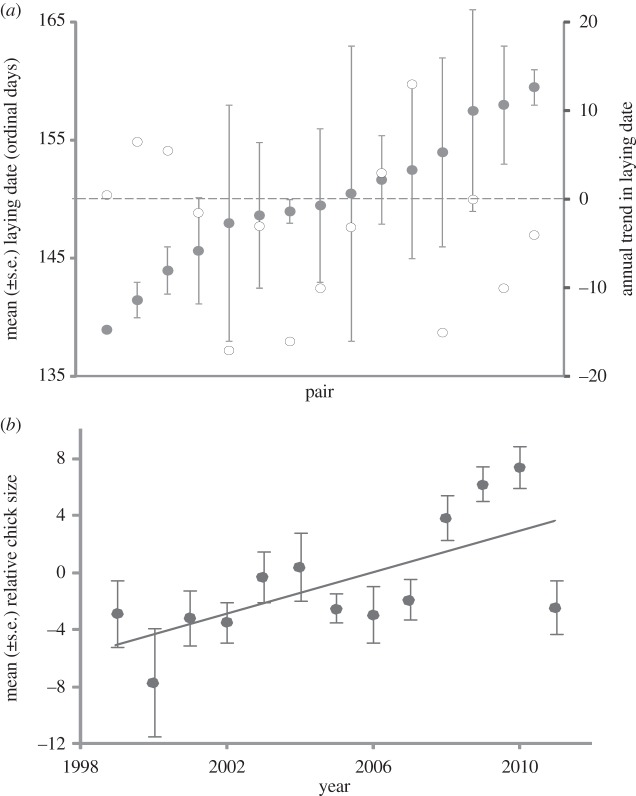
(*a*) Laying dates of 15 pairs of individually marked black-tailed godwits recorded in between 2 and 4 years, between 2001 and 2012 (filled circles, ordered from earliest to latest) and the rates of change (open circles, days per year) in laying among these pairs, and (*b*) annual variation in the average relative size of black-tailed godwit chicks (residual variation from a model of total head length in relation to ordinal date of measurement *y* = 0.72*x* − 1445, *r*^2^ = 0.44, *p* < 0.015) hatched in different years.

**Figure 4.**
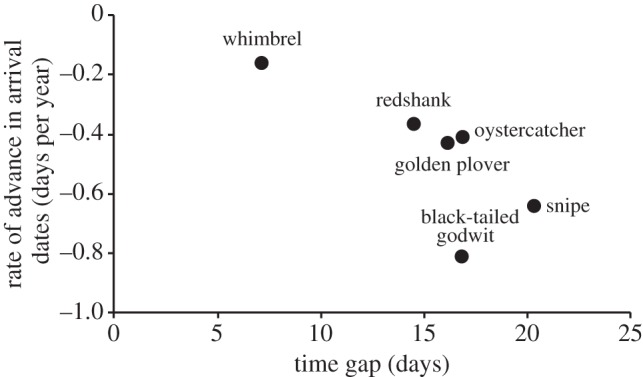
The association between the rate of advance in arrival dates into Iceland of six species of wader (whimbrel, *N. phaeopus*; redshank, *T. totanus*; oystercatcher, *H. ostralegus*; golden plover, *P. apricaria*; black-tailed godwit, *L. limosa* and snipe, *G. gallinago*) and the gap between arrival dates and laying dates (*r*_s_ = −0.81, *p* = 0.05)

Carry-over effects of early life conditions could extend to influence migration timing at recruitment. If early hatching confers benefits of early subsequent migration, changes in nest-laying dates could influence the distribution of arrival dates in populations. Godwits arrive in Iceland between mid-April and mid-May, and nests can be laid from mid-May until mid-June. This large (three to four weeks) interval between arrival and laying provides an opportunity for pairs to respond to annual variation in the timing of local environmental conditions. Pairs of individually marked godwits for which nests have been located in multiple years have low repeatability of laying dates (repeatability = −0.03, *F*_14,19_ = 0.92, *p* = 0.56) and their laying dates have advanced significantly in recent years and are significantly earlier in warmer springs (figure 3*a* and table 1*b*). In addition, the size of chicks caught on any given date throughout Iceland has increased over the last 13 years and is greater in years with warmer mean temperatures in May (*y* = 0.3*x* − 20.9, *r*^2^ = 0.46, *p* < 0.012), again suggesting that hatching dates are advancing (and/or that chick growth rates are increasing) and the frequency of early fledged chicks is increasing (figure 3*b*). If advances in timing of migration are related to advances in nest-laying dates and associated benefits of early hatching, the greatest advances in migratory timings would be expected in species with a greater capacity to advance nest-laying. Across six wader species breeding in Iceland, rates of advance in arrival dates are significantly lower for species with smaller time-gaps between arrival and laying, with the shortest arrival–laying gap occurring in the species (whimbrel) that travels furthest (to sub-Saharan Africa) and has advanced migratory timings the least (figure 4).

#### (ii) Demographic changes associated with migration timing

Changes in the mortality patterns of early arriving recruits could drive population-level advances, for example if more benign weather conditions in recent years increased survival rates of early arriving recruits. However, figure 2*b* suggests both an increase in the frequency of early arriving recruits and a decrease in the frequency of later arriving recruits (recruits hatched from 2007 onwards all arriving before day 115), thus this mechanism would also require recruits arriving later (when conditions are generally more benign) to be subjected to increasing mortality rates.

#### (iii) Environmental determination of migration timing in the recruitment year

Recruiting individuals may respond to weather conditions during migration in the year in which they recruit, resulting in greater numbers of earlier arriving recruits in warmer years. However, as individual arrival dates are highly consistent between years (figure 2*a* and table 1*a*), this mechanism could only operate during the year of recruitment, and arrival dates of the 46 known-age individuals are not significantly related to the NAO index in March (*r*^2^ = 0.005, *p* = 0.63) or April (*r*^2^ = 0.0001, *p* = 0.89), or to mean daily temperature in Iceland during April (*r*^2^ = 0.06, *p* = 0.72) in their likely year of recruitment (second calendar year).

## 4. Discussion

Advances in the timing of spring migration have been reported for many bird species in recent decades, and migratory species have contributed greatly to understanding of the effects of climate change on phenology [7]. However, identification of the mechanisms driving these phenological changes has remained elusive despite evidence of links between changing migration patterns and population declines [15]. The main mechanisms that have been proposed to explain these population-level advances are individual plasticity in timing of migration (individuals advancing their migration in years with better conditions) and microevolutionary responses (adaptation to changing conditions resulting in changes in the frequency of individuals with different migratory timings) [7,17]. Here, we show that individuals are highly consistent in their timing of migration, and that advances in population-level arrival dates are a consequence of new recruits to the population arriving earlier now than in previous years. This mechanism for advancing migratory timings requires neither individual flexibility in annual timing of migration nor changes in gene frequencies. Instead, the distribution of arrival dates within a population shifts through generational changes in migratory timings.

Other studies that have tracked individuals over repeated migrations also consistently report high levels of individual repeatability in timing of migratory movements across taxa [24–28], suggesting that individual plasticity in timing of migration is unlikely to be a common driver of population-level advances in migration. Migratory species typically show very high levels of fidelity to breeding and wintering locations, and the benefits of site-fidelity have been demonstrated empirically and conceptually [29,30]. As the benefits of prior knowledge apply both to known locations and to known times of use of those locations, the selection pressures driving site-fidelity might also be expected to drive this widely observed time-fidelity. If population-level advances in the timing of migration are not the result of individual plasticity in migration timing, changes in the frequency of individuals with different timings must be involved.

Changes in the numbers of individuals with differing arrival dates within a population could result from adaptive selection for earlier arrival. However, similar advances in arrival timings have been reported in species with very different life-history strategies and potential rates of adaptive evolution, suggesting that environmentally induced responses are more likely than microevolutionary adaptations [31]. Environmentally induced advances in arrival dates of recruits could operate through: (i) carry-over effects of changing natal conditions, (ii) changing patterns of mortality of individuals with differing arrival times, or (iii) arrival times being initially determined by conditions in the year of recruitment and individuals repeating those timings thereafter.

In this system, carry-over effects of changing natal conditions may be most likely, as nesting dates of Icelandic godwits are advancing, and previous studies have shown that individuals on earlier breeding schedules tend to occupy better quality wintering locations from which arrival in spring is earlier [10,32–34]. If these strong seasonal links are established through advantages accrued from earlier hatching, such as increased time to fuel and improve body conditions prior to migration and/or earlier departure for winter grounds, and associated benefits, for example increased probability of travelling in adult-dominated migratory flocks [35], advances in nesting dates could increase the frequency of early arriving recruits. Alternatively, timing of hatching could have a more direct relationship with subsequent timings of migration of individuals, for example through the use of environmental cues that vary seasonally, although this would require an endogenous link between cues experienced at hatching and during spring migration some nine to 10 months later at the earliest.

Changes in laying dates in response to environmental conditions on the breeding grounds (such as increased rates of vegetation growth providing nest cover earlier in the season and/or earlier emergence of invertebrate prey in recent, warmer years), and subsequent earlier arrival of recruits hatched earlier in season, could also explain the widely reported differences between short- and long-distance migrants in shifts in arrival [2,5,17]. Long-distance migrant species typically arrive on the breeding grounds later than short-distance species, and the consequent shorter gaps between arrival and laying (figure 4) will limit their capacity to advance laying dates. Migratory timings of long-distance migrants may therefore be constrained by their capacity to advance laying dates, rather than by limited awareness of conditions on the breeding grounds, or constraints imposed by winter conditions and the costs of migration. Populations with greater proportions of recruits would also be expected to have greater capacity for advancing arrival dates, and thus rates of advance in migration timings may vary in relation to life-history strategy, and may be greater in populations experiencing increased productivity and recruitment. Advancing arrival dates of new recruits may therefore be a common mechanism driving population-level shifts in migration timing, and identifying the mechanisms influencing recruit arrival patterns is likely to be key to understanding the links between migration phenology and population change.
